# Ethnobotanical and antimicrobial study of some selected medicinal plants used in Khyber Pakhtunkhwa (KPK) as a potential source to cure infectious diseases

**DOI:** 10.1186/1472-6882-14-122

**Published:** 2014-04-04

**Authors:** Nadeem Khan, Arshad Mehmood Abbasi, Ghulam Dastagir, Abdul Nazir, Ghulam Mujtaba Shah, Mohammad Maroof Shah, Munir H Shah

**Affiliations:** 1 Department of Environmental Sciences, COMSTAS Institute of Information Technology, 22060 Abbottabad, Pakistan; 2 Department of Botany, University of Peshawar, 25120 Peshawar, Pakistan; 3 Department of Botany, Hazara University Mansehra, 21300 KPK, Mansehra, Pakistan; 4 Department of Chemistry, Quaid-i-Azam University Islamabad, 45320 Islamabad, Pakistan

**Keywords:** Ethnobotanical, Antimicrobial, Medicinal plants, Infectious diseases, Pakistan

## Abstract

**Background:**

Present investigation deals with antimicrobial screening of ten medicinally important plants used by the inhabitants of district Haripur, Khyber Pakhtunkhwa (KPK) for different infectious diseases.

**Methods:**

Aqueous, n-hexane and ethanolic extracts of each plant were tested for their antimicrobial activity against both Gram positive and Gram negative strains of bacteria, as well as strain of yeast. Agar well diffusion and broth dilution methods were used to determine the antimicrobial activity of different plant extracts.

**Results:**

The results indicated that all plants exhibited antimicrobial activity against one or more test pathogens. Interestingly, extracts of three plants showed strong and broad spectrum activity as compared to rest of the extracts which demonstrated the moderate activity. On the whole ethanolic extracts exhibited maximum antimicrobial effect than their corresponding aqueous and n-hexane extracts, when compared with standard antibiotics i.e., Streptomycin and Tetracycline. Among various extracts, only ethanloic extract of *Azadirachta indica* and aqueous and ethanolic extracts of *Eucalyptus globulus* and *Bergenia ciliata* and ethanolic extract of *Punica granatum* were found to have potentially promising activity against test microorganisms.

**Conclusion:**

Different plant extracts show promising antimicrobial activity justifying their usage in traditional medicines. This study will be continued to identify more plants with potential antimicrobial components.

## Background

There is an increase interest to explore the secret of traditional herbal remedies based on information collected from local residents and traditional practioners in different parts of the world [1,2]. Chemical and biological investigations of folk medicinal plants with the reputation of being curative have provided the world with many of clinical drugs of today [3]. It has been reported that at least 119 compounds derived from 91 plant species can be regarded as important drugs currently in use and that 77% of them were derived from traditional medicines [4]. The search for new antibacterial agents, in particular, has increased in the last decade mainly because of the increase in bacterial infections especially in countries with poor populations and more so because of bacterial resistance to current antibiotics [5]. Bacterial infections have also been implicated in complication of chronic conditions, especially transplants, cancer, and AIDS because of weakened immunity [6,7].

Studies also claimed that some plants, which are already used as traditional medicine, possess antimicrobial properties against bacteria, fungi, and viruses [8,9] and preparation from such plants considered to be effective against diseases of microbial etiology like small pox, tuberculosis, typhoid and diphtheria etc. [10-12]. Since most of the infectious diseases are commonly treated with antibiotics and some antibiotics like Penicillin is added to items such as chewing gums, mouthwashes and toothpastes. Due to this indiscriminate use of antimicrobial drugs, have lead to the problem of drug resistance [13,14]. Furthermore, the evolution of new strains of disease causing agents is of great concerns to the global health community [15]. In addition to this problem, antibiotic are sometimes associated with adverse effects on host which include, hypersensitivity, depletion of guts and mucosal microorganism, immune suppression and allergic reaction. Furthermore, antibiotics can also be responsible to kill the useful microflora with in the human body [16-20]. It was also found that production of synthetic drugs results into the pollution of natural resources and ultimately leads to activation of antibiotic resistant genes in bacterial population in the environment [21]. All these factors associated with the use of synthetic drugs, has always been one of the driving force that encourage the researchers to search for the safe and economic alternatives from bio resource. One approach is to screen the local medicinal plants for possible antimicrobial properties.

In countries where the infectious diseases are prevalent, there is a need to develop some medicine of plant origin against persisting infectious diseases, which may be comparable to modern medicines and antibiotics. Medicinal plants used in the traditional medicines offer a great reservoir for the discovery of new plants having antimicrobial properties comparable to antibiotics used in modern medicines. Since almost all the antimicrobial agents are being imported and by considering the availability of medicinal plants in these countries, a lot of foreign exchange may be saved. In addition the cost of treatment is steadily increasing and it is becoming unaffordable by common user. Therefore, development of therapeutic agents from our own indigenous resources will be of great help.

In traditional medicines some of the indigenous medicinal plants including *Calotropis procera*, *Artemisia* spp. *Neolitsea chinensis*, *Melia azedarach*, *Azadirachta indica*, *Eucalyptus globulus*, *Punica granatum*, and *Nigella sativa* have been claimed to exert curative effect in the diseases caused by bacterial species. These plants are available in different regions of Pakistan (KPK, North of Punjab and Azad Jammu and Kashmir). All these plants are used in traditional medicines by traditional healer, local inhabitants and herbal practioners in different regions of the country to cure a variety of diseases [22].

The main objective of the present study is to evaluate *in vitro* antimicrobial effects of different medicinal plants based on the information collected from the local residents for the isolation and development of new broad spectrum antimicrobial compounds.

## Methods

### Ethnobotanical survey and collection of plant material and its processing

Periodic surveys were made in the remote areas of district Haripur, KPK, Pakistan during spring and summer. Information regarding the medicinal uses of different plants was collected through interviews with local people and traditional practitioners. Each interview followed a semi-structured questionnaire designed to obtain the following information: local names, parts of plant utilized and their medicinal uses. The sample from each plant was pressed, dried and mounted on herbarium sheets for identification. The plant identification and verification was carried in the Herbarium of Quaid-i-Azam University Islamabad, Pakistan and National Herbarium, National Agriculture and Research Council, Islamabad, Pakistan. The voucher specimen for each plant was submitted in the herbarium of Quaid-i-Azam University, Islamabad, Pakistan.

### Preparation of extracts

Preparation of extracts and antimicrobial assays were conducted in Drugs Control and Traditional Medicine Division, National Institute of Health, Islamabad, Pakistan. The plant material was collected, washed (rhizome and bark) to scrap off the sand or mud. The bark and rhizomes were then cut into smaller pieces and then allowed to dry in shad for 25–30 days. Similarly, all the plant material collected was dried in shade, powdered in an electric grinder. Dried plant material (bark, rhizomes, leaves, seeds and fruits) were divided in lots of 25 g each for extraction. 25 g of ground plant material was extracted successively with distilled H_2_O in Soxhelt extraction apparatus. All these extracts were collected separately and dried in EYELA vacuum rotary evaporator under reduce pressure at 70°C. To evaporate the last traces of H_2_O a water bath is used as a heating source. Here other two portions of ground material (25 g each) were macerated each with absolute ethanol and n-hexane (250 ml) for 8–10 days with regular stirring after 24 hrs. All these extracts were then filtered through Whatmann filter paper No.1. The filtrates were evaporated to a thick residue at 30°C and reduce pressure using EYELA vacuum rotary evaporator. The last traces of ethanolic extracts were evaporated on water bath. However, n-hexane extracts were just evaporated to a thick residue. The powdered aqueous and ethanolic extracts were then dissolved in their respective solvents in a proportion of 50 mg/ml. The concentration of reference antibiotics i.e., Streptomycin and Tetracycline was 100 μg/ml.

### Microbial strains tested

The following strains of bacteria were used: *Bacillus subtilis* ATCC # 6633, *Staphylococcus aureus* ATCC # 6538 (Gram positive strains), *Escherichia coli* ATCC # 10536, *Klebsiella pneumoniae* ATCC # 10031, *Salmonella typhi* ATCC # 19430, and *Shigella dysentriae* ATCC # 11835 (Gram negative strains). The yeast strain used in this study was *Sacchromyces cerevisiae* ATCC # 9763. The tested strains were obtained from Microbiology Laboratory, Drugs Control and Traditional Medicine Division, National Institute of Health, Islamabad, Pakistan. The microorganisms were grown overnight at 37°C in 2% Nutrient Agar (Merk Germany) at pH 7 except yeast which was grown at 30°C. Their sensitivity to the reference antibiotics was checked. Streptomycin and Tetracycline (Sigma, USA) were used for this purpose.

### Preparation of inocula

The inocula were prepared by inoculating a loop of each bacterial strain from a 24 hrs old culture into a sterile nutrient broth aseptically. The culture was allowed to grow for 24 hrs in a shaking incubator at 37°C. The overnight culture is taken and checked until the visible turbidity is equal or greater than that of 0.5 McFarland standards (Pro-Lab Diagnostics) at 560 nm using UV-Visible spectrophotometer (IRMECO UV–VIS U2020, Germany). Sterilized nutrient broth is used as blank. If the absorbance is higher, then the culture is diluted with sterilized nutrient broth and absorbance is noted again. The standardized cultures were used for further analysis.

### Determination of antimicrobial activity

The antibacterial tests were performed using agar well diffusion method [23]. Agar plates were prepared by using sterile Mueller-Hinton (MH) agar (Bio Lab). Bacterial cultures of standardized cultures were prepared by adding the seed culture in the autoclaved agar medium followed by pouring into petri plates. The wells were made with 8 mm sterile cork borer. 50 μl of each extract (50 mg/ml) was added in the pre labelled wells together with water, n-hexane and ethanol as negative control and Streptomycin and Tetracycline as positive control. Both reference antibiotics were used in the concentration of 100 μg/ml. The diffusion of extracts was allowed for 1 hr at room temperature on a sterile bench. The plates were then sealed with Parafilm X and incubated for 24 hrs at 37°C. However, in case of *S. cerevisieae* the plats were incubated for 48 hr at 30°C. The plates were observed for the presence of inhibition of bacterial growth and that was indicated by clear zone of inhibition of bacterial growth around the wells. The size of zone of inhibition was measured in milli meters (mm). Minimum Inhibitory Concentration (MIC) was determined as described by Sahin et al. [24] with little modification. Briefly the sterile broth media (5 ml) was added in to sterile screw cap test tubes followed by the addition of 50 μl diluted microbial suspension in the test tubes. Initially, 1000 μl of each plant extract (50 mg/ml) was added to the corresponding tube. The dilutions for each extract were performed at a final concentration of 2.0, 4.0, 6.0, 8.0 and 10.0 mg/ml. For each dilution 1000 μl of extract was added to corresponding microbial strain and each extract was assayed twice. The extracts in simple broth were used as negative control to ensure medium sterility while the microbial suspensions served as positive control to control the adequacy of the broth for bacterial growth. The test tubes were put on shaker at 37°C for 24−48 hrs to check the microbial growth. After 48 hrs the growth of microbial strains was measured as a function of turbidity. The dilution that showed no turbidity after 24−48 hr was regarded as MIC of the respective plant extract.

## Results and discussions

The medicinal plants find an extensive use in traditional system of medicines all over Pakistan especially in the rural areas to cure various health problems. The main reason for the popularity of herbal medicines are i) these plants are near to nature, hence more effective than allopathic medicines ii) they are easily accessible iii) are cheaper mode of treatment and iv) show fewer side effects or adverse reactions as compared to modern synthetic drugs. In the present study the efforts were made to investigate the antimicrobial activity of those plant species which are used to treat various infectious diseases like syphilis, leprosy, tuberculosis, pneumonia, whooping cough and urinary tract infections [2]. Long before the invention of synthetic antimicrobial drugs, herbal drugs were used to treat such diseases. More than 100 plants in the indigenous system of medicine possessing antibacterial activity have been evaluated [25].

In the present study a total of ten plant species belonging to eight different families were screened for antimicrobial activity. These plants are used by local inhabitants, traditional healers and herbal practitioners in different sites of the visited areas to treat various ailments. Most of these plant species and their parts used to cure various diseases are taken individually. All these plants were collected from different localities of KPK. The common/local names, botanical names, their families, voucher number, plant part used, and their medicinal uses have been described in Table 1. The plants have been listed in alphabetical order according to their families along with respective voucher number. For antimicrobial activity the extracts of each plant were prepared in three fractions namely, aqueous, n-hexane and ethanolic. The antimicrobial activity of each extract was monitored in concentration of 50 mg/ml. The activity of each plant extract was then compared against the standards i.e., Streptomycin and Tetracycline (100 μg/ml). The samples were run in triplicate to get a complete picture of effectiveness of extracts against each microorganism used in the present study. The effectiveness was measured in the form of zone of inhibition.

**Table 1:** Plant species utilized in this study with their medicinal activities

**Family: plant species voucher number**	**Local name**	**Part used**	**Local medicinal uses**	**Medicinal uses from literature**
Asclepiadaceae: *Caloptropis procera* (Aiton) Dryand ISL3504	Akkh	Leaves	Skin infection, back pain, rheumatism, piles, whooping cough, asthma, and insecticidal. Also used in case of venomous animal bits.	Asthma, cough, skin infections, snake bite, scorpion sting [22]
Asteracea: *Artemisia maritima* L. ISL2385	Tarakha	Arial parts	The fresh leaves are chopped and used to cure earache. Also used as anthelmintic, in dysentery, as stomachic and purgative.	Antiseptic and anti-inflammatory, antimalarial, to kill intestinal worms. Also used to cure boils [26,27]
Lauracea: *Neolitsea chinensis* (Gamble) Chun ISL5509	Maidasek/Gwain	Bark	Used in bone fracture and dysentery. It is also used to cure external and internal injuries, rheumatism and sprain. Also used to get relief from back bone pain.	Bark is demulcent, astringent and used in diarrhoea, and dysentery. Also considered as tonic [28]
Meliacea: *Azadirachta indica* A. Juss ISL4529	Neem	Leaves	Used in fever, diabetes, skin infection, dysentery and wound healing. It is also used in foot rots, ulcer washing, to purify blood and to cure malaria. It is also used as carminative.	The leaves are used for acne treatment [29]
Meliacea: *Cedrela toona* Roxb. Ex Rottler ISL4657	Neem	Leaves	Used in fever, diabetes, skin infection and dysentery. It is also sued in wound healing and ulcer washing.	Fever, diabetes, dysentery, blood diseases, skin diseases (allergy and pimples), ulcer and wound healing [22]
Meliacea: *Melia azedarach* L. ISL5723	Dreik	Leaves	Malarial fever, eye diseases, piles, body swelling and head ache. It is also used to purify blood and as carminative. The leaves extract is used to reduce heat effect in summer.	Malarial fever, piles, eye ache, headache, swelling, wounds, blood purification, fever and cough [22,30],
Myratacea: *Eucalyptus globulus* Labill. ISL3341	Safeda	Leaves	Locally it is used to cure flu.	Antidiabetic [31]
Punicacea: *Punica granatum* L. ISL2781	Anar/Daroona	Rind of fruit	It is used in piles, diarrhoea, dysentery, whooping cough, fever and in blood purification. It is also used in stomach disorder, jaundice, toothache and vomiting. Also used to stop bleeding from nose.	Used in diarrhoea, dysentery, piles, diabetes, intestinal worms, fever, blood purification, whooping cough, cooling, indigestion, stomach disorder, liver and intestinal inflammation, jaundice, vomiting, mouth gums, toothache. Dried fruit in bolus form for removal of intestinal helminthes [22,30,32]
Ranunculacea: *Nigella sativa* L. ISL8101	Kalongi	Seeds	The seeds are used as stimulant, digestive and expectorant. It is also used in jaundice, dysentery, diarrhoea, and whooping cough.	The paste prepared by mixing ground seeds in water is used for the treatment of boils [29]
Saxifragacea: *Bergenia ciliata* (Haw.) Sternb. ISL2197	Budpiah	Rhizome	It is used in washing ulcer, to cure back bone and in wound healing. Also used in dysentery and to cure piles. The decoction is used as appendicitis.	Ulcer, back pain, piles, dysentery, and external or internal wounds. Used as tonic and in muscular disorders. Anticancerous drug. Externally used as wound as antiseptic [22,33,34]

The extracts were tested against Gram positive and Gram negative strains of bacteria and the yeast by agar diffusion method, measuring inhibition zone. This method gives the maximum growth conditions for all organisms and avoids the problems of sterilizing plant extracts prior to testing [35]. Table 2 represents the antimicrobial activity (zone of inhibition) of different plant extracts against respective microbial strains.

**Table 2:** Antimicrobial activities (in terms of inhibition zone in mm) where bacterial growth was inhibited by plant extracts

**Plant species**	**Fractions**	**Antimicrobial activity against**						
		***B. subtilis***	***K. pneumonia***	***S. aureus***	***E. coli***	***S. typhi***	***S. dysentriae***	***S. cerevisiae***
*Calotropis procera*	Aqueous	-	-	-	-	-	-	-
	n-Hexane	-	-	-	-	-	-	-
	Ethanol	15.0 ± 0.25	0	14.1 ± 0.25	125 ± 0.20	13.6 ± 0.25	15.2 ± 0.20	14.4 ± 0.264
	Tetracyclin	36.2 ± 1.00	33.6 ± 0.85	32.7 ± 0.90	37.4 ± 0.55	33.8 ± 0.55	34.5 ± 0.72	38 ± 0.152
	Streptomycin	33.4 ± 0.40	32 ± 0.20	34.3 ± 0.55	35.2 ± 0.26	36.2 + 0.10	36 ± 1.00	35.2 ± 0.251
*Artemisia maritima*	Aqueous	-	-	-	-	-	-	-
	n-Hexane	-	-	-	-	-	-	-
	Ethanol	12.2 ± 0.25	12.4 ± 0.37	15.1 ± 0.36	12.8 ± 0.25	13.70 ± 0.41	14.3 ± 0.30	15.6 ± 0.40
	Tetracyclin	36.2 ± 1.00	33.6 ± 0.85	32.7 ± 0.90	37.4 ± 0.55	33.8 ± 0.55	34.5 ± 0.72	38 ± 0.15
	Streptomycin	33.4 ± 0.40	32 ± 0.20	34.3 ± 0.55	35.2 ± 0.26	36.2 + 0.10	36 ± 1.00	35.2 ± 0.25
*Neolitsea chinensis*	Aqueous	-	-	-	-	-	-	-
	n-Hexane	-	-	-	19.36 ± 0.25	17.7 ± 0.26	-	14.86 ± 0.11
	Ethanol	20.1 ± 0.25	-	18.6 ± 0.23	20.0 ± 0.25	19.5 ± 0.20	19.3 ± 0.05	18.7 ± 0.15
	Tetracyclin	36.2 ± 1.00	33.6 ± 0.85	32.7 ± 0.90	37.4 ± 0.55	33.8 ± 0.55	34.5 ± 0.72	38 ± 0.15
	Streptomycin	33.4 ± 0.40	32 ± 0.20	34.3 ± 0.55	35.2 ± 0.26	36.2 + 0.10	36 ± 1.00	35.2 ± 0.25
*Azadirachta indica*	Aqueous	-	-	-	-	-	-	-
	n-Hexane	-	-	-	-	-	-	-
	Ethanol	22.5 ± 0.40	-	17.5 ± 0.30	23.8 ± 0.20	23.9 ± 0.05	19.6 ± 0.15	21.8 ± 0.11
	Tetracyclin	36.2 ± 1.00	33.6 ± 0.85	32.7 ± 0.90	37.4 ± 0.55	33.8 ± 0.55	34.5 ± 0.72	38 ± 0.15
	Streptomycin	33.4 ± 0.40	32 ± 0.20	34.3 ± 0.55	35.2 ± 0.26	36.2 + 0.10	36 ± 1.00	35.2 ± 0.25
*Cedrela toona*	Aqueous	-	-	-	-	-	-	-
	n-Hexane	-	-	-	-	-	-	-
	Ethanol	15.4 ± 0.11	-	13.4 ± 0.25	14.5 ± 0.25	-	-	15.2 ± 0.36
	Tetracyclin	36.2 ± 1.00	33.6 ± 0.85	32.7 ± 0.90	37.4 ± 0.55	33.8 ± 0.55	34.5 ± 0.72	38 ± 0.15
	Streptomycin	33.4 ± 0.40	32 ± 0.20	34.3 ± 0.55	35.2 ± 0.26	36.2 + 0.10	36 ± 1.00	35.2 ± 0.25
*Melia azedarach*	Aqueous	-	-	13.7 ± 0.40	-	-	-	-
	n-Hexane	-	-	-	-	-	-	-
	Ethanol	-	-	-	-	-	-	-
	Tetracyclin	36.2 ± 1.00	33.6 ± 0.85	32.7 ± 0.90	37.4 ± 0.55	33.8 ± 0.55	34.5 ± 0.72	38 ± 0.15
	Streptomycin	33.4 ± 0.40	32 ± 0.20	34.3 ± 0.55	35.2 ± 0.26	36.2 + 0.10	36 ± 1.00	35.2 ± 0.25
*Eucalyptus globulus*	Aqueous	24.1 ± 0.15	28.4 ± 0.30	23.8 ± 0.15	20.0 ± 0.20	18.8 ± 0.17	21.5 ± 0.25	27.5 ± 0.15
	n-Hexane	14.2 ± 0.32	14.5 ± 0.61	12.7 ± 0.25	14.7 ± 0.30	13.2 ± 0.35	15.7 ± 0.25	15.2 ± 0.15
	Ethanol	29.36 ± 0.15	28.73 ± 0.30	26.96 ± 0.15	19.36 ± 0.40	23.6 ± 0.36	25.86 ± 0.11	27.16 ± 0.20
	Tetracyclin	36.2 ± 1.00	33.6 ± 0.85	32.7 ± 0.90	37.4 ± 0.55	33.8 ± 0.55	34.5 ± 0.72	38 ± 0.15
	Streptomycin	33.4 ± 0.40	32 ± 0.20	34.3 ± 0.55	35.2 ± 0.26	36.2 + 0.10	36 ± 1.00	35.2 ± 0.25
*Punica granatum*	Aqueous	14.4 ± 0.32	19.8 ± 0.15	18.7 ± 0.25	13.8 ± 0.11	15.5 ± 0.25	12.6 ± 0.20	15.8 ± 0.15
	n-Hexane	-	-	-	-	-	-	-
	Ethanol	22.5 ± 0.25	23.2 ± 0.20	23.8 ± 0.11	21.5 ± 0.37	23.8 ± 0.15	22.6 ± 0.36	23. 6 ± 0.20
	Tetracyclin	36.2 ± 1.00	33.6 ± 0.85	32.7 ± 0.90	37.4 ± 0.55	33.8 ± 0.55	34.5 ± 0.72	38 ± 0.15
	Streptomycin	33.4 ± 0.40	32 ± 0.20	34.3 ± 0.55	35.2 ± 0.26	36.2 + 0.10	36 ± 1.00	35.2 ± 0.25
*Nigella sativa*	Aqueous	-	-	-	-	-	-	-
	n-Hexane	-	-	-	-	-	-	-
	Ethanol	17.5 ± 0.30	16.0 ± 0.05	18.9 ± 0.15	17.5 ± 0.25	19.7 ± 0.15	15.8 ± 0.11	-
	Tetracyclin	36.2 ± 1.00	33.6 ± 0.85	32.7 ± 0.90	37.4 ± 0.55	33.8 ± 0.55	34.5 ± 0.72	38 ± 0.15
	Streptomycin	33.4 ± 0.40	32 ± 0.20	34.3 ± 0.55	35.2 ± 0.26	36.2 + 0.10	36 ± 1.00	35.2 ± 0.25
*Bergenia ciliata*	Aqueous	15.4 ± 0.26	19.5 ± 0.15	23.7 ± 0.15	23.5 ± 0.32	20.0 ± 0.25	28.6 ± 0.15	23.8 ± 0.15
	n-Hexane	-	-	-	-	-	-	-
	Ethanol	22.830.15	18.6 ± 0.15	24.0 ± 0.10	23.7 ± 0.25	22.8 ± 0.15	29.4 ± 0.47	23.4 ± 0.26
	Tetracyclin	36.2 ± 1.00	33.6 ± 0.85	32.7 ± 0.90	37.4 ± 0.55	33.8 ± 0.55	34.5 ± 0.72	38 ± 0.15
	Streptomycin	33.4 ± 0.40	32 ± 0.20	34.3 ± 0.55	35.2 ± 0.26	36.2 + 0.10	36 ± 1.00	35.2 ± 0.25

±: mean standard deviation of three replicates; −: no zone of inhibition.

The MIC values of the different extracts from various plant species against all tested microorganisms were observed (Table 3). In this case, only those extracts which inhibited the growth of microbial strains in agar well diffusion method were subjected to MIC evaluation. The MIC values of all extracts correlated to the screening test results. It was found that *K. pneumonia* and *S. cerevisiae* were the most susceptible microorganisms with the lowest MIC values of aqueous and ethanolic extracts of *E. globulus* (less than 2 mg/ml). The ethanolic extract of *E. globulus* displayed the same MIC values against *B. subtilis,* and *S. aureus.* However, n-hexane extract of *E. globulus* exhibited the least activity against *S. aureus* (more than 10 mg/ml). Similar trend was found in the *S. dysentriae* when it was subjected to aqueous and ethanloic extract of *B. ciliata*. The ethanolic extracts of *B. ciliata* also exhibited the lowest MIC values against *E. coli* and *S. aureus.* On the other hand, ethanloic extract of *A. maritima* is least effective against *S. cerevisiae* i.e., more than 10 mg/ml. Same behaviour was found in the ethanolic extracts of *C. toona* against *S. aureus* and *E. coli*. Similarly *E. coli* and *S. dysentriae* were least susceptible to aqueous extract of *P. granatum*. Rest of the tested plant extracts exhibited the activity between the concentrations of 2–10 mg/ml (Table 3).

**Table 3:** Minimum Inhibitory Concentration (MIC) values (mg/ml) of different plant extracts against tested microorganisms

**Plant species**	**Extracts**	**MIC (mg/ml) against different microorganism**						
		***B. subtilis***	***K. pneumoniae***	***S. aureus***	***E. coli***	***S. typhi***	***S. dysentriae***	***S. cerevisiae***
*Calotropis procera*	Ethanol	8	-	8	8	8	6	8
*Artemisia maritima*	Ethanol	10	10	8	>10	8	8	6
*Neolitsea chinensis*	n-Hexane	-	-	6	8	-	-	>10
	Ethanol	4	-	6	4	6	6	6
*Azadirachta indica*	Ethanol	2	-	6	2	2	6	4
*Cedrela toona*	Ethanol	8	-	>10	>10	-	-	10
*Melia azedarach*	Ethanol	4	-	8	2	2	6	4
*Eucalyptus globulus*	Aqueous	2	<2	2	4	4	2	<2
	n-Hexane	8	10	>10	10	8	8	10
	Ethanol	<2	<2	<2	4	2	2	<2
*Punica granatum*	Aqueous	10	6	8	>10	8	>10	10
	Ethanol	4	2	4	2	<2	4	<2
*Nigella sativa*	Ethanol	10	8	8	10	6	10	-
*Bergenia ciliata*	Aqueous	8	4	2	<2	4	<2	2
	Ethanol	2	6	<2	2	2	<2	4

Key-: − not tested; > more than; < less than.

The results obtained in the present study revealed that absolute ethanolic extract of *C. procera* showed broad spectrum activity against all the test microorganisms except *K. pneumoniae. S. dysentriae* was the most susceptible microorganism to ethanolic extract with MIC value of 6.0 mg/ml while the growth of other microorganisms was inhibited at MIC value of about 8.0 mg/ml. The antimicrobial activity found in the present investigation is in agreement with the findings of [36] who determined the antimicrobial activity of leaves and latex of *C. procera* against a wide range of microorganisms*.* The aqueous and ethanolic extract of latex of *C. procera* was also found to be effective against *B. subtilis*, *S. aureus*, *E. coli*, and *K. pneumoniae.* It was found that the chloroform, ethyl acetate, n-hexane, methanol and aqueous extracts of another species of *Calotropis* i.e., *C. gigantia* (L.) R. Br. inhibited the growth of different strains of carcinogenic bacteria [37]. It was found that ethanolic extract of aerial parts of *A. maritima* exhibited moderate and broad spectrum activity against all the test microorganisms used in the present study with MIC value ranged from 6.0 mg/ml (*S. cerevisiae*) to more than 10.0 mg/ml for *E. coli*. However, both n-hexane and aqueous extracts of *A. maritima* did not show any activity. This positive response of ethanolic extract of *A. maritima* on different strains of Gram positive and Gram negative strains of bacteria is in accordance with the results evaluated by [38]. However, it was found that 50% ethanolic extract of aerial parts of *A. maritima* did not exhibit any activity against *S. aureus, B. subtilis, E. coli*, and *S. typhi.* This difference in activity might be due to the percentage of ethanolic extract used to measure the antimicrobial activity. The present results are also in agreement with the findings of [5] who found that ethanolic extract of leaves of *A. nilagirica* possesses promising activity against the several clinical pathogens.

Present study reveals that ethanolic extract of the bark of *N. chinensis* has exhibited good and broad spectrum activity against various pathogenic organisms used in the present study except *K. pneumonia* while n-hexane extract exhibited narrow spectrum activity against *E. coli*, *S. typhi* (Gram negative) bacteria and *S. cerevisiae* (Table 2). However, its aqueous extract did not show any activity against any of the tested microorganism. *B. subtilis* and *E. coli* were the most susceptible to ethanolic extract of *N. chinensis* bark (MIC value 4.0 mg/ml). On the other hand *S. cerevisiae* was the least susceptible (MIC value more than 10.0 mg/ml) to n-hexan extract. Other finding supporting the present results is of [39] who reported that 50% ethanolic extract of another species of the same genus i.e., *N. sebifer*a responded positively against *B. subtilis*, *S. aureus*, *E. coli*, and *S. typhi.* The narrow spectrum activity of n-hexane extract of *N. chinensis* against *E. coli* and *S. dysentriae* (Gram negative) bacteria only (Table 2) might be due to solubility of some active constituents in n-hexane which eventually showed this activity.

The *A. indica* is widely used as an insecticidal by the local residents of the visited areas. The ethanolic extract of leaves inhibited the growth of all microorganisms used in the present investigation. The most sensitive microorganisms were *B. subtilis*, *E. coli* and *S. typhi* with MIC value of 2.0 mg/ml and minimum activity was recorded against *S. aureus* and *S. dysentriae* (6.0 mg/ml). On the other hand the aqueous and n-hexane extracts were totally ineffective. *C. toona* is locally used as an alternative of *A. indica*, but the ethanolic extract of *C. toona* leaves is effective against only *B. subtilis*, *S. aureus, E. coli* and *S. cerevisiae* while found ineffective against all the other test microorganisms used in the study (Table 2). Similarly, the aqueous and n-hexane extracts did not show any activity. The results indicated that as for as antimicrobial activity is concerned, the ethanolic extracts of *A. indica* leaves exhibited the strongest antimicrobial activity as compared to *C. toona* though both plants are used for the same purposes in different localities. In case of *M. azedarach* the activity was found only in aqueous extract against *S. aureus* while the ethanolic and n-hexane extracts were totally ineffective in the present investigation (Table 2). The present study strengthens the findings of [16] who showed that absolute ethanolic extracts of bark and leaves of *A. indica* collectively had good activity against *S. cerevicea* and *B. subtilis*. However, [40] could not find any such activity who subjected the ethanolic extract of *A. indica* in various concentrations i.e., 100%, 75%, 50%, 25% and 10% against *Bascillus* spp and *E. coli*. The suppressed activity might be due to the negligible amount of active constituents at the time of collection as compared to present findings. The chloroform extract of *A. indica* was also found to be effective against *S. aureus, Streptococcus pyogens, E. coli* and *Pseudomonas aureginosa*[41]. The methanol extract of *A. indica* was also reported to be effective against *Vibrio cholerae* which is a causative agent of cholera [42].

The leaves of *E. globulus* are used as expectorant, stimulant, antiseptic, carminative, whereas the volatile oil is said to be antimalarial and disinfectant. The aqueous and ethanolic extracts of *E. globulus* leaves exhibited the strongest and the broad spectrum activity against all the test microorganisms where the MIC values are even less than 2.0 mg/ml against different tested pathogens (Table 3). Present study revealed that n-hexane extract of *E. globulus* also has mild antimicrobial activity against all the test microorganisms. These results supported the findings of [43] who tested the antibacterial activity of *E. maidenni* against *K. pneumoniae*. This broad spectrum activity in the present study is also in accordance with the results of [44] who analysed the essential oil composition of 12 species of *Eucalyptus* and tested against *E. coli, S. aureus* and *S. cerevisiae*. It was found that essential oil obtained from *E. globules* and *E. camaldulensis* is effective against *E. coli* and *S. aureus*[45]*.*

The present study revealed that the aqueous and ethanolic extracts of *P. granatum* rind have a good deal of antimicrobial activity. Both the extracts exhibited broad spectrum activity, although ethanolic extract has stronger effect than aqueous fraction (Table 3), while n-hexane extract did not inhibit the growth of any organism (Table 2). This study supported the findings of [46] who found that petroleum ether extract of *P. granatum* had exhibited significant antibacterial activity against *S. dysentreae* and *E. coli*. It was recorded [47] that boiling water extract of *P. granatum* exhibited marked activity against *S. typhi*. The various results supporting these finding are of [48,49]. The work of [50] also supporting these findings. The methanolic extract of fruit peels of *P. granatum* was also effective against *Listeria monocytogenes*, *S. aureus*, *E. coli* and *Yersinia enterocolitica*[51].

The present study revealed that ethanolic extract of *N. sativa* seeds was effective against all tested microorganisms except *S. cerevisiae*, the yeast. On the other hand, n-hexane extract of *N. sativa* seeds was ineffective against all the tested microorganisms in the present study. The work of [52] showed the same behaviour of 90% ethanolic extract of *N. sativa* seeds against *S. typhi, S. dysentriae, Vibrio cholera* and *S. aureus*. Diethyl ether extract of *N. sativa* seeds was found to be effective against *S. aureus*, *E. coli* and *Pseudomonas aeruginosa*[53]. The ethanolic extract was also found to be effective against *Proteus vulgaris* and *Pseudomonas aeruginosa*[16]. The aqueous and ethanolic extracts of *B. ciliata* rhizome exhibited marked and broad spectrum antimicrobial activity against all the test microorganisms used in the present study (Table 2). The highest activity was recorded in the aqueous extract against *E. coli* and *S. dysentriae*. Similar trend was found in the ethanolic extract against *S. aureus* and *S. dysentriae* (Table 3). The present findings are in agreement with the results of [54] who found that various extracts of roots and leaves of *B. ciliata* had promising activity against *B. subtilis*, *Bacilliusm egaterium* and *Pseudomonas aeruginosa*.

In the present study it was found that the absolute ethanolic extract of *A. maritima*, ethanolic extract of leaves *A. indica*, aqueous and ethanolic extracts of *E. globules* and *B. ciliata* rhizome, and ethanolic extracts of *P. granatum* rind inhibited the growth of all microorganisms used in the present investigation (Table 2). Furthermore, the inhibition zone (value) showed by the positive control was significantly smaller compared to those displayed by aqueous and ethanolic extracts of *E. globulus*, ethanolic extracts of *P. granatum* rind, aqueous and ethanolic extracts *B. ciliata* against tested microorganisms. Similar trend was recorded for MIC values of these extract (Table 3). This suggests that the bacterial species are more susceptible to these extracts compared with the other extracts from different plant parts investigated in the present study. The variations of the present results with the previous findings are not unexpected as phyto-constituents are known to vary with ecological factors [55] seasonal variation [56], the type of the solvent, and presence of different chemicals [41] which may or may not be effective against the test microorganisms. The antimicrobial components also change with the age of the plants [57]. Sometimes the compositions of the chemicals change with the orientation of the plant parts. The environmental factors like excessive rain fall or drought enhance the quality of active compounds or diminish it [58].

**Figure 1:**
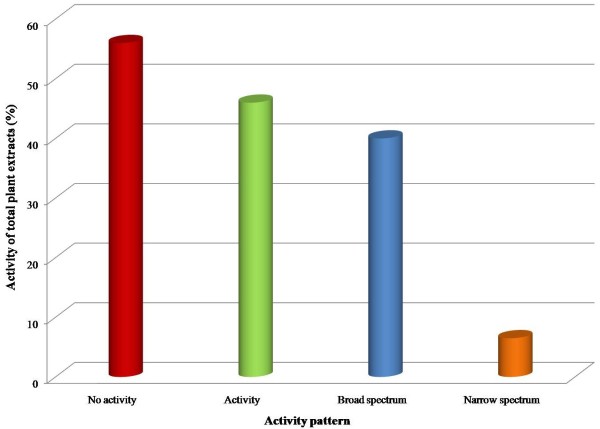
Antimicrobial activity of total plant extracts.

**Figure 2:**
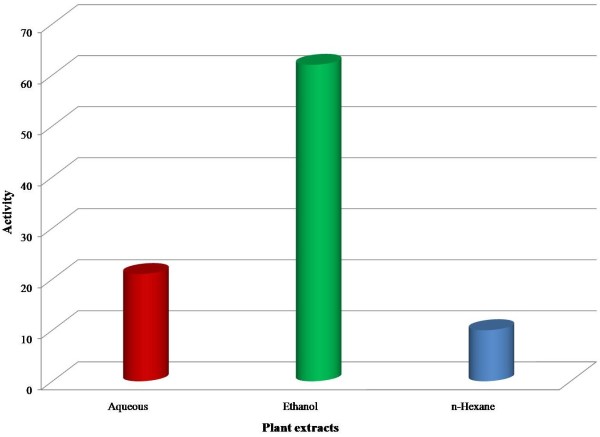
Efficiency of three different solvents used for the extraction of different plant parts to study antimicrobial activity.

It has been evaluated that sixteen plant extracts out of a total of thirty (53%) of different plant parts did not exhibit antimicrobial activity against one or more test microorganism used in the study (Table 2). Out of thirty plants extracts ten plant extracts (40%) i.e., the ethanolic extracts of *C. procera, A. maritima, N. chinensis, A. indica, C. toona*, and that of *N. sativa* inhibited the growth of both Gram positive and Gram negative strains of bacteria i.e., showed broad spectrum activity. Similarly, the aqueous and ethanolic extracts of *E. globulus, P. granatum* and *B. ciliata* also exhibited the stronger and broad spectrum activity. Two plants extract (6.5%) i.e., n-hexane fractions of *N. chinensis* and aqueous extract of *M. azedarach* leaves were found to have narrow spectrum activity. Figure 1 indicates the pattern of antimicrobial activity form different plant extracts against various microorganisms used in the study. In general, ethanolic extract exhibited the highest degree of antimicrobial activity as compared to aqueous and n-hexane extract fractions (Figure 2). This suggests that ethanol is the better solvent to extract active constituents from the plant material than the other solvents used. The Streptomycin and Tetracycline were used as the reference antibiotics in this study. Both have exhibited the antimicrobial activity against all the test microorganisms used in the present investigation. It was found that Tetracycline showed slightly stronger activity against the test microorganisms than Streptomycin. On the other hand all the three solvents remained ineffective in their contact form as they were used as control in each experiment. An interesting finding in the present study is that as for as solvent is concerned the ethanolic extract exhibited the maximum activity as compared to aqueous and n-hexane fractions.

The present study shows that plants possess antimicrobial activity which in the extracted form can be utilized successfully to treat infectious diseases and protect the host from microbial infections. In this regards the plants used in the traditional system of medicine, offer a great reservoir for the discovery of not only antimicrobial drugs but some other drugs as well. Many biological and pharmaceutical groups have already begun to perform the laboratory work in a meaningful way on herbal remedies like the development of analytical methodology from common herbal remedies [59,60] and the toxicological evaluation of phytomedicine compounds [61,62].

## Conclusion

The results obtained in the present investigation have very promising antimicrobial activities and they can be used in the treatment of infectious diseases. The results obtained in the present study justify the use of these medicinal plants to cure various infectious diseases in the rural areas of Pakistan. Furthermore, it is desirable to make in depth studies of these plants for the isolation and characterization of antimicrobial compounds to adjudicate the veracity of the claim.

## Competing interests

The authors declare that there are no conflicts of interest.

## Authors’ contributions

NK has carried out the main experimental work, AMA, AN, GD and GMS have participated in sampling and ethnobotanical data collection, while MMS and MHS critically evaluated the manuscript. All authors read and approved the final manuscript.

## Pre-publication history

The pre-publication history for this paper can be accessed here:

http://www.biomedcentral.com/1472-6882/14/122/prepub
